# Coal Workers’ Pneumoconiosis–Attributable Years of Potential Life Lost to Life Expectancy and Potential Life Lost Before Age 65 Years — United States, 1999–2016

**DOI:** 10.15585/mmwr.mm6730a3

**Published:** 2018-08-03

**Authors:** Jacek M. Mazurek, John Wood, David J. Blackley, David N. Weissman

**Affiliations:** 1 Respiratory Health Division, National Institute for Occupational Safety and Health, CDC.

Coal workers’ pneumoconiosis (CWP) is a preventable occupational lung disease caused by inhaling coal mine dust that can lead to premature* death (*1*,*2*). To assess trends in premature mortality attributed to CWP (*3*), CDC analyzed underlying^†^ causes of death data from 1999 to 2016, the most recent years for which complete data are available. Years of potential life lost to life expectancy (YPLL) and years of potential life lost before age 65 years (YPLL_65_)^§^ were calculated (*4*). During 1999–2016, a total of 38,358 YPLL (mean per decedent = 8.8 years) and 2,707 YPLL_65_ (mean per decedent = 7.3 years) were attributed to CWP. The CWP-attributable YPLL decreased from 3,300 in 1999 to 1,813 in 2007 (p<0.05). No significant change in YPLL occurred after 2007. During 1996–2016, however, the mean YPLL per decedent significantly increased from 8.1 to 12.6 per decedent (p<0.001). Overall, CWP-attributable YPLL_65_ did not change. The mean YPLL_65_ per decedent decreased from 6.5 in 1999 to 4.3 in 2002 (p<0.05), sharply increased to 8.9 in 2005, and then gradually decreased to 6.5 in 2016 (p<0.001). Increases in YPLL per decedent during 1999–2016 indicate that over time decedents aged ≥25 years with CWP lost more years of life relative to their life expectancies, suggesting increased CWP severity and rapid disease progression. This finding underscores the need for strengthening proven prevention measures to prevent premature CWP-associated mortality.

The National Vital Statistics System’s multiple cause-of-death data during 1999–2016 were analyzed to examine CWP mortality. For this analysis, CWP deaths were identified from death certificates listing the *International Classification of Diseases, Tenth Revision* (ICD-10) code J60 (coal workers’ pneumoconiosis) as the underlying cause of death. Because CWP is entirely attributable to occupational exposure (*1*), only deaths of persons aged ≥25 years were considered. Years of potential life lost to life expectancy (YPLL) and before age 65 years (YPLL_65_) were calculated for each decedent. Time-trends in death rates^¶^ (per 1 million population), age-adjusted to the 2000 U.S. standard population and YPLL/YPLL_65_, were assessed (*5*). Information on decedents’ usual industry and occupation** was coded^††^ in accordance with the U.S. Census 2000 Industry and Occupation Classification System.

During 1999–2016, 4,344 decedents aged ≥25 years had CWP assigned as the underlying cause of death, accounting for 38,358 YPLL (mean per decedent = 8.8). Among these decedents, 369 (8.5%) were aged 25–64 years, accounting for 2,707 YPLL_65_ (mean per decedent = 7.3) (Table). Overall, CWP deaths among U.S. residents aged ≥25 years significantly decreased (73%), from 409 in 1999 to 112 in 2016 (Table) (Figure 1). The decline was steeper during 1999–2008 (p<0.01) than during 2008–2016 (p<0.001). CWP deaths among U.S. residents aged ≥65 years decreased 77%, from 389 in 1999 to 88 in 2016. The decline was steeper during 1999–2008 (p<0.001) than during 2008–2016 (p<0.001). Among U.S. residents aged 25–64 years, there was no significant change in the number of CWP deaths during 1999–2016 (Table).

**TABLE:** Years of potential life lost to life expectancy (YPLL) and before age 65 years* (YPLL_65_) for decedents aged ≥25 years with coal workers’ pneumoconiosis,^†^ by sex, race, state of residence, year of death, and industry and occupation^§^ — United States, 1999–2016

Characteristic	All deaths				Deaths at age <65 years			
	No. (%)	Age-adjusted^¶^ rate per million	YPLL	Mean YPLL per decedent	No. (%)	Age-adjusted^¶^ rate per million	YPLL_65_	Mean YPLL_65_ per decedent
**Total**	**4,344 (100.0)**	**1.18**	**38,358**	**8.8**	**369 (100.0)**	**0.11**	**2,707**	**7.3**
**Sex**								
Men	4,292 (98.8)	3.00	37,498	8.7	353 (95.7)	0.20	2,512	7.1
Women	52 (1.2)	0.02	860	16.5	16 (4.3)	Unreliable**	195	12.2
**Race**								
White	4,208 (96.9)	1.28	37,211	8.8	357 (96.5)	0.11	2,605	7.3
Black	124 (2.9)	0.41	1,009	8.1	10 (2.9)	Unreliable	83	8.3
Other^††^	12 (0.3)	Unreliable	138	11.5	2 (0.6)	Unreliable	19	9.5
**State**								
Alabama	47 (1.1)	0.84	359	7.6	—^§§^	—	—	—
Arizona	19 (0.4)	Unreliable	136	7.2	—	—	—	—
Arkansas	18 (0.4)	Unreliable	174	9.7	—	—	—	—
California	34 (0.8)	0.08	387	11.4	—	—	—	—
Colorado	32 (0.7)	0.66	212	6.6	—	—	—	—
Florida	81 (1.9)	0.28	571	7.0	—	—	—	—
Georgia	13 (0.3)	Unreliable	120	9.2	—	—	—	—
Illinois	82 (1.9)	0.53	705	8.6	—	—	—	—
Indiana	61 (1.4)	0.81	472	7.7	—	—	—	—
Kentucky	554 (12.8)	10.55	6,422	11.6	95 (25.7)	1.91	650	6.8
Maryland	15 (0.3)	Unreliable	114	9.8	—	—	—	—
Michigan	34 (0.8)	0.27	229	6.7	—	—	—	—
Missouri	12 (0.3)	Unreliable	78	6.5	—	—	—	—
New Jersey	14 (0.3)	Unreliable	104	7.4	—	—	—	—
New Mexico	39 (0.9)	1.72	269	6.9	—	—	—	—
New York	13 (0.3)	Unreliable	83	6.4	—	—	—	—
North Carolina	40 (0.9)	0.36	388	9.7	—	—	—	—
Ohio	156 (3.6)	1.02	1,166	7.5	—	—	—	—
Oklahoma	10 (0.2)	Unreliable	102	10.2	—	—	—	—
Pennsylvania	1,360 (31.3)	6.96	9,109	6.7	24 (6.5)	0.14	172	7.2
South Carolina	20 (0.5)	0.35	268	13.4	—	—	—	—
Tennessee	99 (2.3)	1.35	953	9.6	12 (3.3)	Unreliable	98	8.2
Texas	18 (0.4)	Unreliable	199	11.1	—	—	—	—
Utah	45 (1.0)	2.10	352	7.8	—	—	—	—
Virginia	558 (12.8)	6.37	6,103	10.8	84 (22.8)	0.94	649	7.7
West Virginia	892 (20.5)	33.37	8,543	9.6	86 (23.3)	3.62	507	5.9
Wyoming	14 (0.3)	Unreliable	131	9.4	—	—	—	—
All other states^¶¶^	64 (1.5)	—	671	—	68 (18.4)	—	—	—
**Year**								
1999	409 (9.4)	2.31	3,300	8.1	20 (5.4)	0.13	129	6.5
2000	389 (9.0)	2.18	3,044	7.8	19 (5.1)	Unreliable	136	7.2
2001	367 (8.4)	2.04	2,858	7.8	12 (3.3)	Unreliable	65	5.4
2002	354 (8.1)	1.94	2,741	7.7	21 (5.7)	0.14	90	4.3
2003	318 (7.3)	1.70	2,513	7.9	18 (4.9)	Unreliable	99	5.5
2004	292 (6.7)	1.52	2,375	8.1	20 (5.4)	0.09	192	9.6
2005	270 (6.2)	1.41	2,155	8.0	21 (5.7)	0.14	187	8.9
2006	266 (6.1)	1.34	2,259	8.5	18 (4.9)	Unreliable	160	8.9
2007	209 (4.8)	1.06	1,813	8.7	16 (4.3)	Unreliable	142	8.9
2008	183 (4.2)	0.90	1,756	9.6	21 (5.7)	0.12	196	9.3
2009	206 (4.7)	0.96	2,162	10.5	32 (8.7)	0.15	271	8.5
2010	213 (4.9)	1.01	2,024	9.5	23 (6.2)	0.12	187	8.1
2011	160 (3.7)	0.72	1,560	9.8	18 (4.9)	Unreliable	138	7.7
2012	158 (3.6)	0.69	1,634	10.3	21 (5.7)	0.09	139	6.6
2013	150 (3.5)	0.66	1,485	9.9	18 (4.9)	Unreliable	119	6.6
2014	155 (3.6)	0.64	1,769	11.4	27 (7.3)	0.11	188	7.0
2015	133 (3.1)	0.54	1,497	11.3	20 (5.4)	0.09	114	5.7
2016	112 (2.6)	0.44	1,413	12.6	24 (6.5)	0.11	155	6.5
**Industry**								
Coal mining	560 (75.7)	—	5,415	9.7	63 (74.1)	—	417	6.6
Construction	31 (4.2)	—	306	9.9	—	—	—	—
Nonpaid worker or nonworker including at home	14 (1.9)	—	161	11.5	—	—	—	—
All other industries	135 (18.2)	—	1,350	10.0	22 (25.9)	—	197	9.0
**Occupation**								
Mining machine operators	504 (68.1)	—	4,822	9.6	52 (61.2)	—	365	7.0
Electricians	16 (2.2)	—	152	9.5	—	—	—	—
Laborers and freight, stock, and material movers	14 (1.9)	—	147	10.5	—	—	—	—
Construction laborers	13 (1.8)	—	135	10.4	—	—	—	—
First-line supervisors or managers of construction trades and extraction workers	13 (1.8)	—	134	10.3	—	—	—	—
Homemakers	13 (1.8)	—	143	11.0	—	—	—	—
Driver-sales workers and truck drivers	11 (1.5)	—	170	15.5	—	—	—	—
All other occupations	156 (21.1)	—	1,532	9.8	33 (38.8)	—	249	7.5

**Source:** National Vital Statistics System; https://wonder.cdc.gov/ (for rates) and multiple cause-of-death data, National Center for Health Statistics, CDC (for YPLL and YPLL_65_).

**Abbreviations:** YPLL = years of potential life lost to life expectancy; YPLL_65_ = years of potential life lost before age 65 years.

* YPLL to life expectancy was a sum of the differences between the age at death and life expectancy for each decedent internally adjusted by race and sex (https://www.cdc.gov/nchs/products/life_tables.htm). YPLL_65_ was a sum of the differences between 65 years and the age at death for each decedent.

^†^ Decedents whose death certificates listed the *International Classification of Diseases, Tenth Revision* (ICD-10) code J60 (coal workers’ pneumoconiosis) as the underlying cause of death.

^§^ Industry and occupation data available for 740 (94.6%) of 782 CWP deaths among U.S. residents aged ≥25 years and for 85 (89.5%) of 95 CWP deaths among U.S. residents aged 25–64 years that occurred in 26 states during 1999, 2003, 2004, and 2007–2012. https://www.cdc.gov/niosh/topics/noms/default.html.

^¶^ Adjusted to the 2000 U.S. standard population.

** Relative standard error ≥23%; rate considered statistically unreliable. https://wonder.cdc.gov/wonder/help/ucd.html#Unreliable.

^††^ Includes American Indian or Alaska Native and Asian or Pacific Islander.

^§§^ Suppressed because of confidentiality constraints (<10 decedents reported). https://wonder.cdc.gov/wonder/help/ucd.html#Assurance of Confidentiality.

^¶¶^ States with <10 decedents.

**FIGURE 1:**
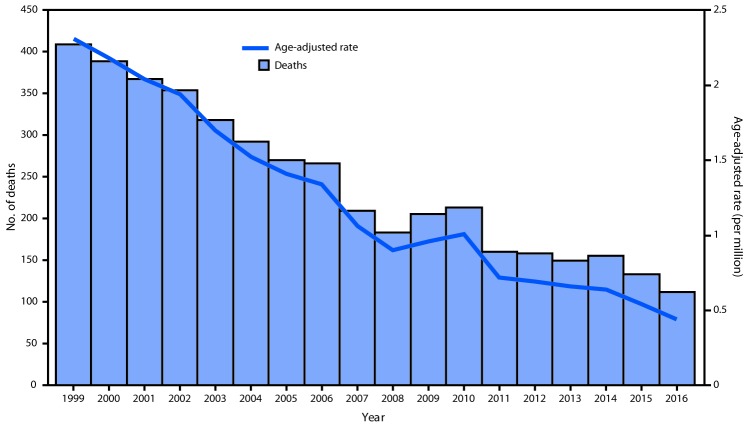
Age-adjusted coal workers’ pneumoconiosis deaths and deaths* per million persons aged ≥25 years with coal workers’ pneumoconiosis,^^†^^ by year of death — United States, 1999–2016 **Source:** National Vital Statistics System. https://wonder.cdc.gov. * Adjusted to the 2000 U.S. standard population. ^†^ Decedents whose death certificates listed the *International Classification of Diseases,* *Tenth Revision* (ICD-10) code J60 (coal workers’ pneumoconiosis) as the underlying cause of death.

Age-adjusted CWP death rates among U.S. residents aged ≥25 years declined 81%, from 2.31 per million in 1999 to 0.44 in 2016 (annual percent change [APC] = -9.0%; 95% confidence interval [CI] = -9.6 to -8.3; p<0.05) (Figure 1). Age-adjusted CWP death rates among residents aged ≥65 years declined 84% from 11.30 per million in 1999 to 1.82 in 2016 (APC = -9.6%; 95% CI = -10.3 to -8.9; p<0.05).

The CWP-attributable YPLL decreased 42.8% from 3,300 in 1999 to 1,413 in 2016 (Table) (Figure 2). The decline was steeper during 1999–2007 (p<0.001) than during 2007–2016 (p<0.05). During 1999–2016, the mean YPLL per decedent increased 55.6%, from 8.1 to 12.6 years per decedent. No significant change in the mean YPLL per decedent was observed during 1999–2003; however, mean YPLL per decedent increased significantly from 2003 to 2016 (p<0.001).

**FIGURE 2:**
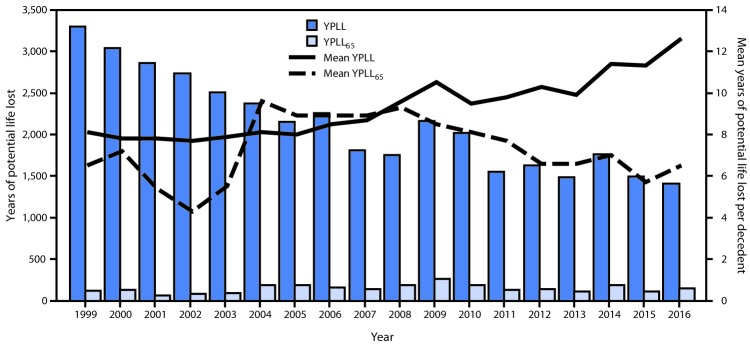
Years of potential life lost to life expectancy (YPLL) and before age 65 years (YPLL_65_) and mean YPLL and YPLL_65_ per decedent for decedents aged ≥25 years with coal workers’ pneumoconiosis,* by year of death — United States, 1999–2016 **Source:** Multiple cause-of-death data, National Center for Health Statistics, CDC. * Decedents whose death certificates listed the *International Classification of Diseases,* *Tenth Revision* (ICD-10) code J60 (coal workers’ pneumoconiosis) as the underlying cause of death.

CWP-attributable YPLL_65_ varied annually, from a high of 271 (mean per decedent = 8.5) in 2009 to a low of 65 (mean per decedent = 5.4) in 2001 (Table) (Figure 2). Overall, no change in the YPLL_65_ from 1999 to 2016 was observed. The time-trend analysis indicates that the mean CWP-attributable YPLL_65_ per decedent aged 25–64 years decreased 34% from 6.5 YPLL_65_ per decedent in 1999 to 4.3 in 2002 (p<0.05), sharply increased 107% to 8.9 in 2005 (p = 0.06), and then gradually decreased 27% to 6.5 in 2016 (p<0.001). In these three respective periods, highest mean YPLLs_65_ per decedent were 7.2 in 2000; 9.6 in 2004, and 9.3 in 2008.

During 1999–2016, ≥10 CWP deaths among persons aged ≥25 years occurred in 27 states. Deaths in Pennsylvania (1,360; 9,109 YPLL; mean per decedent = 6.7), West Virginia (892; 8,543 YPLL; 9.6), Virginia (558; 6,013 YPLL; 10.8), and Kentucky (554; 6,422 YPLL; 11.6) accounted for 77.6% of all decedents and 78.4% of the total YPLL (Table). CWP deaths among persons aged 25–64 years in Kentucky (95; 650 YPLL_65_; mean per decedent = 6.8), West Virginia (86; 507 YPLL_65_; 5.9), Virginia (84; 649 YPLL_65_; 7.7), and Pennsylvania (24; 172 YPLL_65_; 7.2), accounted for 78.3% of all decedents and 73.1% of the total YPLL_65_.

Industry and occupation data were available for 740^§§^ (94.6%) of 782 CWP deaths among U.S. residents aged ≥25 years that occurred in 26 states during 1999, 2003, 2004, and 2007–2012 (Table). By industry, three quarters of deaths occurred among residents who worked in the coal mining industry (560; 75.7%) accounting for 5,415 YPLL (mean per decedent = 9.7). By occupation, approximately two thirds of deaths occurred among mining machine operators (504; 68.1%) accounting for 4,822 YPLL (mean per decedent = 9.6). Remaining CWP deaths were associated with 68 other industries and 79 other occupations.

## Discussion

CDC’s National Institute for Occupational Safety and Health (NIOSH) examined information on CWP deaths reported during 1968–2006, which indicated that CWP deaths and annual YPLL_65_ attributed to CWP have been decreasing (*3*). The findings in the current report indicate that CWP deaths among U.S. residents aged ≥65 years continued to decrease during 1999–2016; however, no significant changes in CWP deaths among persons aged 25–64 years and CWP-attributable YPLL_65_ were observed. Furthermore, there was a sharp increase in the mean YPLL_65_ per decedent since 2002, with a peak (9.6 years) in 2004, followed by a continual, albeit slow, decline. Also, while there was a decline in YPLL during 1999–2016, the increase in the mean YPLL per decedent during this period indicates that each year, on average, decedents aged ≥25 years with CWP lost more years of life relative to their life expectancies. These premature deaths are consistent with observed increased severity and rapid progression of disease (*6*–*8*).

The decline in age-adjusted CWP death rates and CWP-attributable YPLL might be explained, in part, by the decline in employment in the mining industry. The growing gap between each decedent’s actual age at death from CWP and his or her life expectancy corroborates recent reports of increasing prevalence and severity of CWP and of rapid disease progression among coal miners (*6*–*8*). In particular, an 8.6-fold increase in the prevalence of progressive massive fibrosis (PMF) from an annual average of 0.37% during 1994–1998 to 3.23% during 2008–2012, was identified among longer-tenured Kentucky, Virginia, and West Virginia underground coal miners participating in the Coal Workers’ Health Surveillance Program (*6*,*7*). Most of the CWP deaths in this report (68%) occurred among mining machine operators. This finding is consistent with a report describing a cluster of PMF cases identified in coal miners at a clinic in Kentucky, which found that a high proportion (76%) of miners reported working as roof bolters or continuous miner operators (*6*). In addition, a recent study of 416 primarily former miners with PMF served by a network of three Black Lung Clinics in Southwest Virginia represents the largest known cluster of PMF reported in the scientific literature; one third of miners with CWP had indications of exceptionally severe and rapidly progressive disease (*9*). Moreover, an increase in lung transplants performed for patients with CWP has been reported during 2008–2014 (*10*).

The findings in this report are subject to at least four limitations. First, CWP diagnosis as the underlying cause of death could not be validated. Some deaths from CWP might have been attributed to other interstitial lung diseases (e.g., idiopathic pulmonary fibrosis) or other chronic diseases (e.g., chronic obstructive pulmonary disease) occurring in coal miners. Second, there is no specific ICD-10 code for PMF to allow better identification of decedents with severe CWP. Third, complete work histories were not available for analyses. Finally, YPLL and YPLL_65_ in this report did not account for reduced quality of life or work years lost attributed to disability from CWP.^¶¶^

In 2014, a new Federal Rule*** on miners’ occupational exposure to respirable coal mine dust was introduced. The rule decreased allowable exposure to respirable coal mine dust, made changes in dust monitoring, and directed NIOSH to expand medical monitoring for coal mine dust lung diseases. CDC provides information about diseases caused by coal mine dust and the Coal Workers’ Health Surveillance Program.^†††^ The continuing occurrence of premature deaths from CWP underscores the need for primary prevention through prevention of exposures to hazardous levels of coal mine dust, secondary prevention through early disease detection and prevention of further hazardous exposures, and tertiary prevention through provision of appropriate medical care to persons with CWP.

**Summary**

**What is already known about this topic?**

Coal workers’ pneumoconiosis (CWP) is a preventable occupational lung disease caused by inhaling coal mine dust; CWP can progress to respiratory failure and premature death.

**What is added by this report?**

During 1999–2016, the mean CWP-attributable years of potential life lost per decedent increased from 8.1 to 12.6 years, likely because of increased severity and rapid progression of CWP.

**What are the implications for public health practice?**

The continuing occurrence of premature deaths from CWP underscores the need for primary prevention by preventing hazardous exposures to coal mine dust, secondary prevention by early disease detection and prevention of further hazardous exposures, and tertiary prevention by providing appropriate medical care to persons with CWP.

## Footnotes

* Early pneumoconiosis (i.e., simple CWP) is often asymptomatic, but the disease can progress to more severe forms associated with substantial impairment, including progressive massive fibrosis. Progression can occur despite cessation of exposure. Progression might be more rapid after high levels of exposure to coal mine dust or if the dust has a high respirable crystalline silica content. There are no accepted specific therapies to prevent progression of CWP.

† Underlying cause of death is defined as “the disease or injury which initiated the chain of morbid events leading directly to death, or the circumstances of the accident or violence which produced the fatal injury.” https://webappa.cdc.gov/ords/norms.html.

§ YPLL to life expectancy may be considered as a loss of years from the overall life span. YPLL to life expectancy was a sum of the differences between the age at death and life expectancy for each decedent, internally adjusted by race and sex (https://www.cdc.gov/nchs/products/life_tables.htm). YPLL_65_ may be considered as a loss of years from a traditional working life. YPLL_65_ were calculated as a sum of the differences between age 65 years and the age at death for each decedent.

¶ The Joinpoint Regression program fits the simplest joinpoint model that the data allow. It determines years when changes in the number of deaths/YPLL/mean YPLL per decedent and the annual percentage change in log-transformed age-adjusted mortality rate occur by performing a sequence of permutation tests using Monte Carlo sampling and the Bonferroni correction for multiple testing. The overall statistical significance level was α = 0.05, with a maximum of three joinpoints and four trend segments allowed.

** Twenty-six states provided data for the years 1999, 2003, 2004, and 2007–2012: Colorado, Florida, Georgia, Hawaii, Idaho, Indiana, Kansas, Kentucky, Louisiana, Michigan, Nebraska, Nevada, New Hampshire, New Jersey, New Mexico, North Carolina, North Dakota, Ohio, Rhode Island, South Carolina, Texas, Utah, Vermont, Washington, West Virginia, and Wisconsin. The state represents the state where the death took place. https://www.cdc.gov/niosh/topics/noms/default.html.

†† https://webappa.cdc.gov/ords/norms-glossary.html#ind-occ.

§§ For 42 residents of these 26 states, deaths occurred in states other than the state of residence that did not code the industry and occupation information.

¶¶ Persons with CWP can live for many years with severely impaired lung function and be unable to work. Coal mining has also been associated with silicosis, mixed dust pneumoconiosis, dust-related diffuse fibrosis, and chronic obstructive pulmonary disease (COPD) including emphysema and chronic bronchitis. COPD might develop independently of CWP.

***
https://www.gpo.gov/fdsys/pkg/FR-2014-05-01/pdf/2014-09084.pdf .

††† https://www.cdc.gov/niosh/topics/cwhsp.
